# Empowering Patients Through Virtual Care Delivery: Qualitative Study With Micropractice Clinic Patients and Health Care Providers

**DOI:** 10.2196/32528

**Published:** 2022-04-27

**Authors:** Lindsay Burton, Kathy L Rush, Mindy A Smith, Selena Davis, Patricia Rodriguez Echeverria, Lina Suazo Hidalgo, Matthias Görges

**Affiliations:** 1 School of Nursing University of British Columbia-Okanagan Kelowna, BC Canada; 2 Kootenay Boundary Patient Advisory Committee & Community Trail, BC Canada; 3 Department of Family Medicine Michigan State University East Lansing, MI United States; 4 Department of Family Practice The University of British Columbia Vancouver, BC Canada; 5 Kootenay Micro Practice Nelson, BC Canada; 6 Department of Anesthesiology Pharmacology & Therapeutics The University of British Columbia Vancouver, BC Canada; 7 Research Institute BC Children’s Hospital Vancouver, BC Canada

**Keywords:** virtual care, micropractice, focus groups, patient portals, COVID-19, family practice, rural care, digital health technology, telehealth

## Abstract

**Background:**

Prior to the wider adoption of digital health technologies during the COVID-19 pandemic, applications of virtual care were largely limited to specialist visits and remote care using telehealth (phone or video) applications. Data sharing approaches using tethered patient portals were mostly built around hospitals and larger care systems. These portals offer opportunities for improved communication, but despite a belief that care has improved, they have so far shown few outcome improvements beyond medication adherence. Less is known about use of virtual care and related tools in the outpatient context and particularly in rural community contexts.

**Objective:**

This study aims to reflect on the opportunities and barriers for sustainable virtual care through an example of a digitally enabled rural micropractice, which has provided 10%-15% virtual care since 2016 and 70% virtual care since March 2020.

**Methods:**

Three focus groups, 1 with providers (physician and medical office manager) and 2 with a total of 8 patients from a rural micropractice in British Columbia, were conducted in November 2020 and December 2020. Virtual care delivery was explored through the topics of communication approach, mixing virtual and in-person care, the practice team’s journey in developing these approaches, and provider and patient satisfaction with the care model. Interviews were transcribed, checked for accuracy against recordings, and thematically analyzed.

**Results:**

Both patients and providers reported ease of communication and high satisfaction. Either could initiate communication, and patients found the ability to share health information asynchronously through the portal allowed time to reflect and prepare their thoughts. Patients were highly engaged and reported feeling empowered and true partners in their health care, although they noted limited care coordination with specialists. The mix of virtual and in-person visits was highly regarded by patients and providers, and patients reported feeling safe and cared for 24/7, although both expressed concern about work spilling into the provider’s home life. The physician worried about missed diagnoses with virtual care. With respect to establishing the micropractice, solutions took about 5 years to optimize, with providers noting a learning curve requiring technical support for both themselves and their patients and a willingness to respond to patient feedback to identify the best solutions. Despite a mature virtual practice, patients reported deferred care due to COVID-19.

**Conclusions:**

The micropractice’s hybrid care model encouraged patients to be true partners in their care and resulted in high patient engagement and satisfaction; yet, success may rely on the patient population being willing to engage and being comfortable with technology. Barriers lie in gaps in care coordination and provider fear that signs or symptoms more evident with an in-person exam could be missed. Even in this setting, deferral of care in light of COVID-19 was present, and opportunities to address care gaps should be sought.

## Introduction

### Background

Virtual care represents any nondirect synchronous or asynchronous interaction between patients or members of their circle of care, using any form of communication or information technology. Virtual care encompasses a range of health care activities enabled by technology, including telehealth (the term commonly used for the delivery of care through video or telephone conferencing with a care provider) [1]. Before wider adoption of digital health technologies during the early phases of the COVID-19 pandemic, applications of virtual care were mostly in the form of virtual walk-in clinics using tools enabling communication between health care providers for specialist visits or for phone or video visits between providers and patients in remote settings [2]. These applications were primarily focused on diagnosis, prevention, and public health [3]. However, the COVID-19 pandemic prompted widespread emergency adoption of virtual care models that were haphazardly implemented, with the primary aim of reducing infection risk but also conserving resources including personal protective equipment [4].

### Virtual Care Opportunities

Patient engagement has not traditionally been a focus of virtual care applications [3]. While there is no strong evidence that educating health care providers in patient self-management approaches will actually improve outcomes [5], patient empowerment appears to improve medication adherence, and patient engagement may lead to better mental and physical health outcomes [6]. Virtual care tools include interconnected personal health records and tethered patient portals, videoconferencing solutions, remote patient monitoring, and wearable technologies for such things as electronic collection of patient-generated data and delivery of patient education; they allow patients and family members to advocate for their needs, including health and wellness and preventative care services. These tools enhance the potential for participation and bidirectional communication, both asynchronously and synchronously, between patients and providers; hence, they offer a mechanism to increase patient engagement and empowerment [7-9]. While there are limited data supporting change in patient health outcomes, a meta-analysis of patient portals tethered to hospital electronic medical records (EMR) found that patient portals improved patient safety, medication adherence, patient-provider communication, and patient engagement, and both patients and providers believed that portals improved patient care [10]. Less is known about best practices for outpatient and community use of virtual care technologies. Although patient portals are becoming more common, only 20% of Canadians say they have accessed their health information through a blend of channels (mostly lab results and not primary care records) [11-13], and availability and use are far from equitable, leading one author to suggest that the digital divide may be yet another social determinant of health [14]. However, a hybrid approach, combining virtual care with a more traditional in-person approach, might enhance access opportunities.

Access to care has traditionally been a challenge in the Canadian rural setting [15]. The COVID-19 pandemic highlighted the opportunities offered by telehealth and virtual care [16], notwithstanding their current limitations. Given the likelihood that virtual care is here to stay, preparing for transition to long-term sustainable implementations should leverage experiences from stakeholders, both before and during the COVID-19 pandemic. This presents an opportunity for the health system to plan, for providers to learn from their peers in identifying the optimal balance between in-person care and virtual care, and for patients to benefit from advanced digital tools and improved access and patient empowerment.

### Micropractice Care Approach

Since the early 21st century, the micropractice model of health care has appeared in the United States, Eastern Mediterranean, and Canada. A clinical microsystem approach advocates for constant communication between health care providers and their patients to understand mutual expectations about care and appreciate the patient’s needs and clinician’s concerns [17]; it does so by focusing on 6 aims of quality improvement: patient safety, clinical care effectiveness, patient focus, timeliness of care delivery, efficiency in care delivery, and equity of access to care [18]. Micropractices, as a patient-focused, quality-assurance, and data-driven care model, may be an efficient and cost-effective model for primary care delivery that allows health care providers to spend more time with their patients and leverage new technologies and approaches of care more easily than regular (larger) practices [19,20]. In a recent systematic review looking at whether clinical microsystems work, only 3 of the 35 papers included in the review targeted primary care [17]. However, the foci of these studies were highly specific to either a chronic condition [21] or to team effectiveness and care coordination activities [22,23], and neither focused on the patient and provider as a core unit of analysis nor a virtual micropractice delivery model in a rural setting.

### Study Aim

The aim of this paper was to explore the opportunities and barriers for sustainable patient-centered virtual care through an example of a rural micropractice, which has provided 10%-15% virtual care since 2016 and 70% virtual care since March 2020. The clinic, as a single-physician, virtually enabled practice pre-COVID-19, offered an opportunity for the researchers to explore a unique model of care during a public health disaster and for community-based providers to learn about digital enablement from the experience of patients and providers in a micropractice.

## Methods

### Location

This qualitative descriptive study targeted a micropractice as part of a larger mixed methods study examining data sharing and personal health records among rural primary care practices in British Columbia, a province in western Canada. The practice is located in a small rural community with a population of about 11,000 people and was identified as a valuable exemplar in its application of digital health technology deserving of further exploration.

### Ethical Approval

Ethical approval was granted by the joint review boards of the University of British Columbia Clinical Research Ethics Board (H19-00958; principal investigator, KLR) and the Interior Health Research Ethics Board. This article follows the Consolidated Criteria for Reporting Qualitative Health Research (COREQ) [24].

### Participant Recruitment

The providers of the micropractice were approached to participate through an email invitation from our team’s digital health liaison. The micropractice providers then sent a broadcast email advertising the study to all portal-registered patients over the age of 18 years. Interested patients contacted the research assistant who obtained informed consent. Patients received a CAD $25 (US $19.93) gift certificate for their participation, and the physician and clinic manager or medical office assistant (MOA) was remunerated commensurate with provincial guidelines for payment of health professionals participating in research.

### Data Collection

A focus group was conducted in November 2020 with the physician and the clinic manager or MOA from the micropractice. Questions asked providers about types of data shared, platforms and tools used for data sharing both pre- and postpandemic, value and trust in patient-generated data, and use of patient-generated data in care decisions. Two focus groups of 4 patients each were conducted in December 2020 following informed consent and completion of a brief survey including demographic information, health status, and use of technology. Focus groups lasted approximately 1 hour and were guided by a semistructured interview guide with questions about types of data or health information shared with their providers; who initiated sharing; problems encountered with electronic data sharing; current and preferred data sharing features, functions, and processes; value of data sharing and sense of partnership with provider; and communication changes since the pandemic. Focus group participants were encouraged to not only address the researcher’s questions but also build constructively on the comments made by other participants.

One experienced team member facilitated each focus group (MG, KLR), with at least 2 other research team members present in each group, to cofacilitate as needed. Each focus group was held on Zoom videoconferencing software (Zoom Video Communications, San Jose, CA). All sessions were audio-recorded, with nonmoderating team members taking notes and asking questions to probe for follow-up details or clarification by relaying them to the focus group moderator via a chat function.

### Data Analysis

Interviews were transcribed automatically using NVivo Transcription (QSR International, Melbourne, Australia), checked for accuracy against the recordings by a research team member, and thematically analyzed [25]. Initial coding was completed according to a priori categories (eg, electronic communication, selection of tools or platforms, and satisfaction). Through team discussion and consensus, a final schema was generated, with themes emerging from the data, to capture (1) learning and evolving the micropractice model, (2) communicating meaningfully, (3) partnering in care, and (4) transition to (increased) virtual care during the COVID-19 pandemic.

## Results

### Participant Characteristics

Eight patients participated in 2 patient focus groups. All (8/8) were Caucasian, most (6/8) were female, median age was 60.5 (IQR 35-76) years, most (6/8) were college or university graduates, and participants had a wide range of incomes. Median distance from the micropractice was 3 (range 1-10) km. These patients had also sought health care from a median of 2 (range 0-6) specialists, but only 2 of the 8 patients had required a hospital admission in the past year. Two participants reported poor health, and 5 patients considered themselves in good or excellent health. All used technology at least some of the time, with 7 considering themselves a regular or frequent user. In addition, the 2 providers of the micropractice clinical team, a physician and the clinic manager or MOA who own the micropractice, participated in a separate provider focus group; due to the small sample size, their characteristics are not described here. The micropractice has a panel of approximately 700 patients; most have access to the electronic tools utilized by the micropractice (only 19 patients do not).

### Themes

Four themes emerged to describe patients’ and providers’ experiences with the micropractice. These included learning and evolving the micropractice model, communicating meaningfully, partnering in care, and transitioning seamlessly to an increased virtual care model during the pandemic. The Results section is organized around these 4 themes.

### Learning and Evolving the Micropractice Model

Both patients and health care providers acknowledged the need for learning to operate in a technology-enabled virtual care model. Providers described a learning curve for both themselves and their patients. For themselves, it was learning over time, through trial and error in using different systems, what constituted the optimal technology and electronic systems to support their practice. The micropractice providers use Med Access EMR (Telus Health, Montreal, Canada) and have been using a virtual attendant, text messaging, and phone-based appointments (RingCentral, Belmont, CA) with online booking (Veribook, Toronto, Canada) since March 2016. They have used videoconferencing visits since 2017 and used RingCentral (RingCentral, Belmont, CA) until mid-2019. They also have a website that explains how the practice works (eg, restricted phone hours but 24/7 messaging). In mid-2019, they centralized and integrated the virtual care options with their EMR, adding a tethered patient portal (Pomelo Health, Montreal, Canada) for videoconferencing visits, secure messaging, appointment booking and reminders, completion of some forms, and broadcast messaging and information sharing; email is only used as a last resort. Patient communication is supported and, in part, triaged by the medical office manager and ultimately addressed by the physician when needed.

Providers described how important it was for clinic personnel to be very comfortable with technology, to have an open mind to ride the learning curve, and to not resort to going back to “what we know” and how they practiced using these digital tools before making them available to their patients. Providers also set up tools and guidance for patients on using digital tools (eg, video appointments) on their clinic website, including FAQs. If needed, the clinic manager provided tool education before appointments, and providers educated patients to appropriate appointment types (eg, virtual vs in-person) to best support their use. Additionally, providers learned about what was optimal through patients’ feedback. Some changes to the practice model were prompted by patient feedback, including negatively perceived online reviews:

Because of that feedback, we decided to change things around with the schedule and the paperwork. So now it looks like I'm more available, even though I'm still working the same hours. [...] We do take the feedback seriously.

It was also noted that some patients may have left the practice as this care model did not suit them. While this practice is located in a small city, access to high-speed internet was not identified as a problem by the providers, who noted that fewer than 3% of their patients were without internet access. Similarly, patients also noted that internet access was not a concern for them.

Providers learned to be proactive and anticipate future challenges and difficulties so they could plan accordingly to remediate them or reduce their negative impact. For example, providers began letting patients know in advance when the clinic would be closed for vacations, and patients spoke of valuing this mass communication to keep them informed and be able to do their own planning. Additionally, providers highlighted the importance of maintaining strong boundaries between personal and work life. Finally, providers highlighted that it was essential to have health care team autonomy and proper compensation for the implementation and delivery of virtual care to make this model sustainable.

The patients in our focus groups speculated that patients likely self-selected for this virtual care model, based on familiarity with technology and a willingness to take an active role in their care. Finally, several patients offered suggestions for improving the technology, for example, to accommodate the visually and hearing impaired (eg, running text beneath a picture) and incorporating additional patient data into clinical care such as step counter (pedometer) results. One patient suggested that use of a dialog box or personal diary between him and the physician might help him decide when a visit is necessary or to avoid putting off needed care. Another patient suggested additional reminders for things like preventive care.

### Communicating Meaningfully

Communication was at the heart of patients’ and providers’ experiences with the micropractice; both described various facets of the communication that characterized their micropractice experience. These included the nature of the communication as promoting dialogue, enhancing data sharing, and including family.

#### Promoting Dialogue

Unique to this practice was the fluid bilateral dialogue that was both synchronous (eg, virtual visits) and asynchronous (eg, email, secure text, appointment booking) that patients found engaging and empowering. One advantage highlighted by both health care providers and patients was that there were no set rules nor conventions about who would initiate a conversation. Sometimes the patient would begin the exchange, and, with the portal, they were not confined to office hours, such as sending pictures of something painful or making appointments in the middle of the night:

The technology is so helpful—it's...all online, and you can book at 2:00 AM—you don't have to wait to call to talk to a receptionist.

At other times, the practice providers would reach out with reminders, follow up on results of tests, or ask patients to schedule an in-person appointment. Patients did not comment on preferences in communication mode, other than preferring the security of the portal compared with email.

The ability to have more of a dialogue-based communication approach made these patients feel more empowered in their care due to the ability to ask questions and send pictures, compared with a physician-driven exchange:

I had booked an in-person appointment for a lump that came up in my back. I was very concerned about that, and so I just added that in the comments because the appointment was so far away. And then I very quickly got a response that was like, we want to talk to you sooner.

I got an infected foot, and I wasn't quite sure what it was, but I didn't know how the heck she was going to decide whether I needed to come in or not unless I took a picture of it.

Patients who liked the asynchronous communication approach described the time it gave them to rephrase their response, rethink their request, and remember answers instead of having to respond on the spot, as when communicating synchronously. Furthermore, one patient noted that there were things that were more easily expressed in writing rather than face-to-face, such as embarrassing conditions or symptoms:

It comes out a lot easier, and I can say more through a message. And then, by the time I get to the face to face, like the embarrassment is all gone.

In contrast, other participants found a relative lack of ease and candor in the virtual setting.

Patients had reasonable expectations for response times, understanding that they would not get a reply on the weekend but expecting text messages to have a slightly shorter response time than portal messages. Patients were oftentimes surprised to get written responses outside regular office hours:

I've gotten replies and emails back like in the middle of the night because she's just kind of up and whatever and she never stops working

...

If there's some sense of urgency, it's not as if she hangs up the computer at 5 o'clock in the afternoon. There tends to be responses at interesting times. So, we feel very safe and comfortable about that.

Despite the value of dialogue for both patients and providers, there was acknowledgement of the impact on providers. Providers acknowledged that it caused a slight blurring of work and home life for them, but the text messaging system, or the “text line,” akin to an office phone line, protected their personal phone number from the patient. Further, patients expressed worry about burdening their physician too much.

#### Enhancing Data Sharing

The integrated, multimodal system and patient portal allowed for communication of a range of types of patient-generated data. Such data included general intake questionnaires (eg, before a pap smear, COVID-19 screening before a visit, new mother, or new patient intake forms), clinical screening tools (eg, Patient Health Questionnaire-9, Generalized Anxiety Questionnaire-7, chronic pain questionnaires, or attention deficit hyperactivity disorder assessment forms), documentation of patient-recorded measurements (eg, blood pressure, heart rates, daily weights, or blood glucose readings), any clinical notes (eg, lab work, investigations, or consults) that the patient might have, pictures from skin lesions, applications for patient benefits, and any appropriate consents (eg, for treatment, requesting medical records, sharing medical information with a caregiver). Health care providers felt that their relationship with their patients allowed them to trust patient-provided information in all but few cases, such as patients with memory limitations or those seeking specific benefits (eg, in disability applications):

If I have an elderly patient with dementia, I'm not going to be asking [that] from that patient—maybe I'm going to be asking their caregiver instead. For the most part, the data that I'm collecting, I feel comfortable trusting it because I know the patient is capable of giving me good information or giving me the right data.

The clinic manager or MOA was responsible for checking the portal and downloading information into the EMR.

Patients and providers found it challenging that platforms for data sharing did not transfer to other care providers to allow for better care coordination. For example, 1 patient noted:

I would love to see more data sharing and more integration. I would love to be able to book an appointment with my specialist the same way but [this particular specialist] doesn’t offer eHealth appointments. It would be amazing if it was all integrated; [clinicians would realize] that I haven't had imaging done in a while.

This might lead to timelier receipt of results:

If those results were available online instead of being mailed to my house [...] when moving around for school, I would not have gotten them months later.

The physician reported frustration over lack of system integration with other providers, resulting in delayed receipt of consult reports from specialists. This caused both patient frustration and resulted in a delayed implementation of specialist recommendations:

Many times, we need to go out of our way and “fish” for them so we can get back to the patients with a plan and update their medical records.

Patients were uniformly positive about this new care delivery system. One patient noted that other health care providers were “way behind” in offering virtual access to care:

The way in which I approach my access to the health care system changed before COVID. I'm noticing that other physicians are way behind—my specialist does not have the same ability to provide me care that they had before the pandemic. They aren't up to speed with how to do this electronically...so I can get better care by following up with

the micropractice provider

#### Inclusion of the Family

Patients appreciated that the virtual care approach enabled family members to be involved more easily. This included granting access to information and communication to local family members, children, or carers, but the system also allowed involvement of family members outside the province to participate in their care:

What's been fascinating about this approach is that my separated spouse in Ontario can participate as well as I can. And that's been incredibly helpful [...]; it provides a level of foundation, a solidity, that I'm not sure we would have had, had there not been this micropractice.

Health care providers illustrated an example in which a very complex patient, who required point-of-care testing, was able to make use of the virtual care technology to receive optimal care with the help of a (distant) family member; this would have been very difficult in a traditional office-based practice setting.

### Partnering in Care

Unique to this practice is patients’ and providers’ shared sense of a partnership and patients feeling empowered to take ownership of their care. Both patients and health care providers liked the micropractice care model:

You know, you get your full time with them, and they're on time, and I think I feel more heard than when I was in the clinic.

They are simply an amazing team, the two of them.

Health care providers felt they provided very patient-centered care, recognizing that patients enjoyed this access to their provider and generally did not abuse it, but they also missed some separation of work from home life due to the 24/7 messaging.

Overall, patients felt that there was a true partnership, and health care providers valued their opinions, whether the appointment was conducted in-person or virtually, with the clinician offering them opportunities to make informed choices. Patients unanimously agreed that this practice approach empowered them and forced them to take greater ownership of their own care:

I have a couple of chronic conditions that I kind of monitor bloodwork for. And I look after myself, and I can do a lot of that kind of self-monitoring. [...] I have the same access to my bloodwork as my physician, so we can both look at it at the same time. I really appreciate that, and it makes me take more responsibility and feel empowered and not helpless.

### Seamless Transition to (Increased) Virtual Care During the COVID-19 Pandemic

Prior to the pandemic, virtual care was used for a substantial portion of patient visits at the micropractice. Prepandemic use eased the care disruption and confusion that occurred with the pandemic shift to a predominant virtual model of care. Patients recognized that virtual care was often the only way they could seek care during the pandemic and acknowledged the benefit of prior familiarity with virtual care (eg, for some mental health concerns). One patient said:

Had we had not been transitioning to this style of medical care when the pandemic hit, I think it would have been way crazy, a lot clumsier, and a lot harder.

Providers similarly lauded being able to seamlessly respond to the virtual shift in care due to the pandemic:

I knew these stories from colleagues having to close the clinics because at the beginning of the pandemic, you couldn't see patients, but we never stopped. We had a video conference. [...] And even though patients were not using it much before, now was the opportunity to really push it and get it to what we envision at some point. And now it's probably past that. [...] We had texting. We had messaging. We have it all in play.

Both patients and providers expressed concerns about not having in-person visits. The health care providers suggested that they sometimes feared not getting the whole picture and missing problems during virtual visits:

Missing like, for example, checking the blood pressure of my patients when they come or maybe looking at moles or maybe listening to hearts. I have found things when they come for some other unrelated issues [...] now that I'm not seeing them that often, how many things am I missing?

These concerns were echoed in patients’ concerns that virtual visits provided fewer opportunities than face-to-face meetings to discuss other issues. These concerns were accentuated during the pandemic. Several patients noted deferring needed care during the pandemic such as follow-up blood tests, with 1 patient mentioning being selective about what to visit the doctor for and not being comfortable in a crowded clinic for minor problems, noting how helpful it was having a virtual care format; 1 patient noted:

I don't want to go into the lab. I don't want to wait in a line up at the hospital for anything. If I go there and I see a bunch of people, I come home. I just don't go. [...] having your doctor appointments electronically, amazing, I never have to have that fear of, you know, waiting in a waiting room full of people.

Patients appreciated not having to spend time sitting in waiting rooms or travelling to a speciality clinic that offers virtual visits, which could be substantial in remote settings (eg, 1 hour to 1.5 hours each way); 1 patient noted:

Reflecting on my previous experience with an in-person clinic, I have had to wait over an hour, and when you're already taking an hour off of work to go to an appointment, to then have to take 2 hours off of work. [...] I've missed several appointments where I've waited as long as I can

and then left

## Discussion

### Principal Findings

This study suggests that the micropractice care approach can enable patients to be true partners in their care and have more meaningful communication with their providers. Patients who participated in this study were highly engaged in their care, felt safe, and were able to initiate care when needed. These patients liked the asynchronous communication encouraged by this practice model as it allows both immediacy and having time to formulate their thoughts. Providers in the study were proud of delivering very patient-centered care, although they acknowledged that it caused a slight blurring of work and home life. Having a virtual care system in place proved advantageous during the pandemic, although patients reported some deferral of care, which must be addressed as the pandemic eases. The care model, however, likely depends on self-selected patients who are technology-capable and are willing to initiate communication through the portal. Identified challenges of this virtually enabled care model includes providers’ fears of missing symptoms and indicators that they may have otherwise noticed during an in-person visit and the time and effort needed to train both patients and providers in the use of digital technology.

### Learning and Evolving the Micropractice Model

Micropractices may allow health care providers to spend more time with their patients and can leverage new technologies and approaches to care more easily than regular (larger) practices [19,20]. Other reported advantages are increased provider satisfaction with their work, including being able to spend more time with their family, but which may come with reduced scope of practice [26]. In contrast, in this work, both health care provider and patients expressed concerns regarding work-life balance for their provider team. In addition, the micropractice team noted that it takes time to find the right systems and there is a digital health learning curve for both patients and providers (eg, requiring technical support). While not identified as an issue in this study, the chasm between those who have internet access to technologies and the digital literacy to work them and those who don’t may to lead to disparities and inequities and warrants further research [27].

Historically, micropractice patients report excellent continuity of care, delivered with high efficiency and low barriers to access, yet their value for enabling patients to manage their care remains less clear [28]. A systematic review of 35 studies evaluating clinical microsystems, of which 18 described general practice clinics, found that implementation of these care models helped develop a patient-centered approach; promoted interdisciplinarity and quality improvement skills; and increased clinical efficiency, patient safety, and patient and clinician satisfaction [17]. A recent UK-based qualitative study of general practitioners practicing in micropractice settings found that, while this care model increased clinician satisfaction, quality improvement efforts focused more on administrative or process metrics than health outcome-focused metrics, and, as such, the value of this approach in improving patient outcomes remains unclear [29].

### Communicating Meaningfully

Care coordination is a challenge in health care due in part to communication barriers between primary care providers and specialists; this was no different for the micropractice providers and their patients who participated in our study. Care coordination strategies may improve health outcomes after hospital discharge [30]; for example, using technology to deliver discharge information was the preferred method for both health care providers and patients [31]. While awaiting province-wide integrated EMRs that allow data sharing, suggestions for improving care coordination include use of smartphone apps [32], patient portals as a communication tool for care coordination [33], and developing middleware solutions for transferring data from personal health records to EMRs [34].

Virtual 2-way patient-provider communication was a valued component of the micropractice we studied, which highlighted the dialogical nature of communication between patients and health care providers who are not constrained by traditional office-hour conventions. This contrasts with concerns raised previously by physicians about the obligation of communicating beyond normal working hours [35] and reflects the patient-centric ethos of the micropractice. The ability of the providers in this study to make themselves more available while working the same number of hours through use of multiple communication modalities allowed patients to share their data and questions at times that were relevant to them (ie, when they were experiencing concerns). The timeliness of the responses contrasts with the long patient waiting times typical of traditional primary care providers [36,37].

### Partnering in Care

Personal health records may increase health-protective behaviors and facilitate a more patient-focused health partnership and social care system [38]; yet, evidence indicates that uptake of patient portals and personal health records is low [39,40]. The majority of studies, however, used patient portals as a supplement to in-person visits, making the portal seem more peripheral to care and potentially discouraging its use. In the micropractice we studied, portal use was patients’ primary mode for accessing care and interacting with their providers; this appeared to facilitate high patient engagement, a behavior also observed in patients in rural New Zealand [41]. Supporting this idea, authors of a systematic review reported patients’ interest in using portals for patient-clinician communication as one of the areas with strongest evidence [42]. Recommendations for patient-provider partnerships in care and to build lasting digitally enabled care models include the use of low-threshold technologies, security and privacy regulations, reimbursement and liability policies, training and awareness of the technology’s limitations, and not completely replacing the role of in-person medicine [9].

### Seamless Transition to (Increased) Virtual Care During the COVID-19 Pandemic

Although the decision for scheduling mostly virtual visits during the pandemic was made by the micropractice team, patients expressed great satisfaction with the virtual care received, in part because they were already familiar with virtual visits and also likely due to their underlying relationship with, and trust in, the micropractice team. A survey of 420 patients attending virtual visits before the pandemic found that over 80% of patients agreed or strongly agreed that their virtual visit was as good as an in-person visit by a clinician [43]. While having a prior relationship with their virtual visit clinician was associated with less comfort and ease with virtual technology [43], here the opposite was found, possibly due to enhanced digital data sharing experienced by micropractice patients. Patients expressed hopes that such health data sharing systems would increase ownership in their care, improve timeliness and efficiency of care delivery, increase care personalization, and lead to safer care [44].

The fear about missed diagnoses during virtual care expressed by the micropractice physicians was also seen in a recent media review, in which health care providers worried that less frequent care, or more impersonal virtual care, might worsen health outcomes [45]. Missed diagnoses during virtual care have not been widely reported; in contrast, a report of tele-ophthalmology during the COVID-19 pandemic indicated appropriate triage and what appeared to be reasonable patient safety, with only 1.5% of virtual visits resulting in an in-person visit within 1 day or 5.4% in a visit within 2 weeks [46].

Despite the value of virtual care, the micropractice team in this study expressed concern that care had been missed. This is consistent with evidence of deferral of primary care in the United States, Canada, and the Netherlands during the COVID-19 pandemic, including concerns about missed referrals and routine care, increasing risk for morbidity and mortality [47,48]. As the acute phase of the COVID-19 pandemic subsides, this issue may resolve; however, health care providers may need to strategize ways to address this issue for future emergencies [49] and plan for catching up on preventive activities such as immunizations [50,51] and cancer screening [52,53].

### Limitations

In this study, we focused on only 1 micropractice and its patients and health care providers, which limits transferability of the results, including the potential to adapt its success to other practices. There is also the possibility of selection bias, whereby those more enthusiastic about the virtual care approach volunteered to participate in focus groups. Yet, this in-depth exploration provides unique insights that might be lost by pooling results with other practices that adopted virtual care during the pandemic. Another limitation might be that the health care providers owned the micropractice; however, the providers were not involved in the study design, collection of patient data, nor the analysis of results. Additionally, they might be more invested in its success and be willing to tolerate hardships more than if they had been employees. As such, additional insights from micropractices that are not owner-operated may be useful to inform the feasibility and utility of larger-scale implementation.

### Future Work

Additional study is needed regarding implementation of digital solutions in primary care practices with respect to different stages of practice and technology readiness for implementation, number of features added separately or together, and the influence on patient outcomes and team-based care. A comparison of the experiences of patients and health care providers in this rural micropractice with rural practices that only adopted virtual care as a response to the COVID-19 pandemic might be insightful to identify additional barriers and opportunities for long-term sustainable implementation of virtual and technologically enhanced care.

### Conclusions

Using focus groups of health care providers and patients of a virtually enabled micropractice, we identified that opportunities of this hybrid care model lie in patients being true partners in their care. This can result in high patient engagement and satisfaction. Yet, the virtual care model needs to take account of less technology-engaged or technology-comfortable patients. Barriers lie in gaps in coordination of care with other practices that are less technology-enabled and provider fear that signs or symptoms more evident with an in-person examination could be missed. Finally, even in this setting, deferral of care occurred during the COVID-19 pandemic, and opportunities to address care gaps, including prevention, should be sought.

## Abbreviations

COREQ: Consolidated Criteria for Reporting Qualitative Health Research
EMR: electronic medical record
MOA: medical office assistant
